# Heart rate variability biofeedback: how and why does it work?

**DOI:** 10.3389/fpsyg.2014.00756

**Published:** 2014-07-21

**Authors:** Paul M. Lehrer, Richard Gevirtz

**Affiliations:** ^1^Department of Psychiatry, Rutgers – Robert Wood Johnson Medical SchoolPiscataway, NJ, USA; ^2^California School of Professional Psychology, Alliant UniversitySan Diego, CA, USA

**Keywords:** heart rate variability, biofeedback, resonance, baroreflex, homeostasis

## Abstract

In recent years there has been substantial support for heart rate variability biofeedback (HRVB) as a treatment for a variety of disorders and for performance enhancement (Gevirtz, 2013). Since conditions as widely varied as asthma and depression seem to respond to this form of cardiorespiratory feedback training, the issue of possible mechanisms becomes more salient. The most supported possible mechanism is the strengthening of homeostasis in the baroreceptor (Vaschillo et al., 2002; Lehrer et al., 2003). Recently, the effect on the vagal afferent pathway to the frontal cortical areas has been proposed. In this article, we review these and other possible mechanisms that might explain the positive effects of HRVB.

## INTRODUCTION

In recent years there has been substantial support for heart rate variability biofeedback (HRVB) for a variety of disorders and for performance enhancement (Gevirtz, 2013). Since conditions as widely varied as asthma and irritable bowel syndrome seem to respond to this form of cardiorespiratory feedback training, the issue of possible mechanisms becomes more salient. The most supported possible mechanism is the strengthening of homeostasis in the baroreceptor (Vaschillo et al., 2002, 2006; Lehrer et al., 2003). Recently, the effect on the vagal afferent pathway to the frontal cortical areas has been proposed. In this article, we review these and other possible mechanisms that might explain the positive effects of HRVB.

**FIGURE 1:**
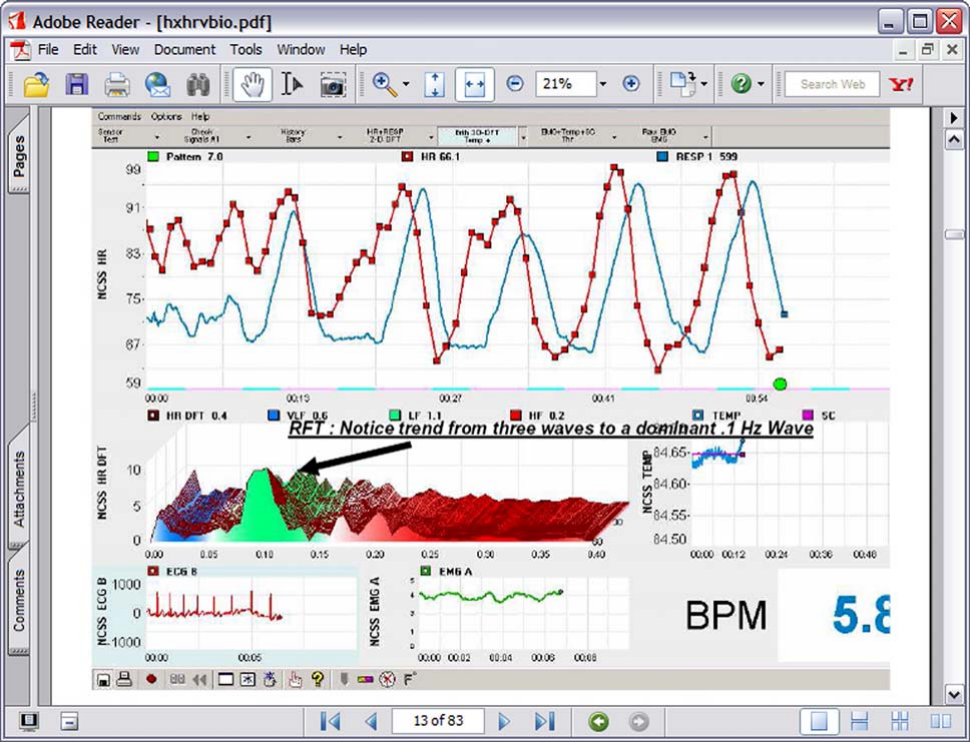
**A typical HRVB screen showing the transition from normal breathing to 7 breaths per minute breathing**.

In the 1990s Lehrer et al. (2000) began experimenting with a form of cardiorespiratory intervention that has subsequently been labeled HRVB, respiratory sinus arrhythmia (RSA) biofeedback, or resonance frequency feedback (RFF). The procedure consists of feeding back beat by beat heart rate data during slow breathing maneuvers such that the participant tries to maximize RSA, create a sine-wave-like curve of peaks and valleys, and match RSA to heart rate patterns. RSA is the heart pattern that occurs when heart rate increases during inhalation and decreases during exhalation. Thus as can be seen in **Figure 1**, the participant uses feedback or a breath pacing device to produce the characteristic maximized RSA.

Gevirtz (2013) recently reviewed all of the available literature on the outcomes of HRVB. He looked at the following application categories: asthma, COPD, IBS, cyclic vomiting, recurrent abdominal pain, fibromyalgia, cardiac rehabilitation, hypertension, chronic muscle pain, pregnancy induced hypertension, depression, anxiety, PTSD, insomnia, and performance^1^. While few areas have extensive support by way of controlled studies, the overall picture seems to be very promising for this intervention. As can be seen, the applications are quite varied. We have begun to explore what physiological and/or psychological mechanisms might be contributing to these positive outcomes.

## MECHANISMS BY WHICH HIGH-AMPLITUDES OF HRV ARE ACHIEVED DURING HRV BIOFEEDBACK

Heart rate variability (HRV) has a complex structure, often referred to as “chaotic,” involving various superimposed oscillation frequencies, non-linearly related to each other (Ivanov et al., 1999; Pikkujamsa et al., 1999). Some processes involved in this pattern are caused by known reflexes, some with modulatory functions, often controlled by different autonomic pathways. These can be described as “negative feedback loops,” which operate as closed loop system components that help maintain allostatic balance, while allowing adaptation to environmental demands (Lehrer and Eddie, 2013). During HRV biofeedback, the amplitude of heart rate oscillations grows to many times the amplitude at rest, while the pattern becomes simple and sinusoidal. This pattern occurs in almost everyone, and is often achievable within a fraction of a minute even in persons who have never previously been exposed to the technique. The mechanism for this effect lies in a confluence of processes: (1) phase relationships between heart rate oscillations and breathing at specific frequencies, (2) phase relationships between heart rate and blood pressure oscillations at specific frequencies, (3) activity of the baroreflex, and (4) resonance characteristics of the cardiovascular system.

## PHASE RELATIONSHIPS BETWEEN HEART RATE OSCILLATIONS AND BREATHING

During normal breathing, one of the many oscillations in heart rate usually occurs at the same frequency as breathing. People often breathe at differing frequencies at different times, and various individuals tend to breathe at different rates. For most people, most of the time, breathing frequency is between 0.15 and 0.4 Hz, or 9 to 24 breaths per minute. The corresponding oscillations in heart rate are called RSA, which can be interpreted as influences of respiration on the sinoatrial node of the heart. The frequency range of 0.15–0.4 Hz is the “high frequency” (HF) band in the HRV spectrum, and spectral amplitude within this range is often used as an index of RSA (Berntson et al., 1997). However, although RSA is often driven by breathing, it also may be influenced by respiratory pacemaker oscillations in the central nervous system, which occasionally differ from actual breathing. These processes might be influenced by external factors (e.g., sudden exercise or stress, sighs, etc.), where both pacemaker and actual respiration may both produce heart rate oscillations. These can sometimes occur at different frequencies and with different patterns, as shown in dissociation between RSA and breathing during mechanical ventilation (Van de Louw et al., 2010), apnea (Passino et al., 1997), and paced breathing (Song and Lehrer, 2003). At resting respiratory rates, the phase relationship between breathing and HR is far from synchronous, such that heart rate increases tend to follow inhalation at about the mid-breath point, and heart rate decreases follow exhalation also at about the mid-breath point (Vaschillo et al., 2002).

Respiratory sinus arrhythmia is known to have important regulatory functions. It controls the rate of gas exchange at the alveoli, such that heart rate tends to be higher when air in the lung is richest in oxygen, and exhalation occurs when carbon dioxide in the lung is highest. It is notable, however, that the partial out-of-phase relationship between heart rate and breathing is *not* the most efficient pattern for gas exchange. Animal experiments by Hayano et al. (1996) in Japan have found that gas exchange at the alveoli is most efficient when heart rate starts increasing at the beginning of inhalation, and starts decreasing just as exhalation starts, i.e., a 0° phase relationship. In these studies, denervated dogs were artificially ventilated, and heart rate oscillations were entirely controlled by a cardiac pacemaker, such that the phase relationship between respiration and heart rate could be experimentally manipulated, in three phase relationships: 0, 90, and 180° (the last of these corresponding to a pattern where heart rate started increasing at the beginning of each exhalation, and started decreasing at the beginning of each exhalation). They measured gas exchange in the alveoli, and found that it was greatest at the 0° phase relationship, at an intermediate level at the 90° phase relationship, and lowest at the 180° phase relationship. Perhaps the function of a partially out-of-phase phase relationship is to allow the greatest degree of flexibility to the organism, such that greater efficiency can be achieved during greater metabolic need, and less during decreased need. Phase relationship studies at various levels of metabolic need have not yet been done, so this interpretation must remain speculative.

Respiratory sinus arrhythmia also can reflect aspects of autonomic function. It is controlled entirely by the vagus nerve, such that vagus nerve outputs to the sinoatrial node primarily occur only during exhalation. Greater vagus nerve traffic will therefore produce greater amplitudes of RSA, such that many scientists equate RSA (or HF HRV) with “cardiac vagal tone,” or parasympathetic influence on the heart (Berntson et al., 1997). However, longer exhalations (Strauss-Blasche et al., 2000) and slower respiration (Eckberg et al., 1985; Grossman et al., 1991; Song and Lehrer, 2003) also may increase RSA amplitude, possibly independently of vagus nerve traffic, since vagus nerve output occurs for relatively longer periods of time with each breath.

Indeed, it has long been known that amplitude of HRV is systematically related to breathing frequency, with higher amplitudes achievable with slower respiration (Eckberg et al., 1985; Brown et al., 1993; Badra et al., 2001; Eckberg, 2003; Song and Lehrer, 2003). However, most studies find that maximum effects usually are achieved when breathing at a rate of approximately 0.1 Hz (six breaths per minute). Working in St. Petersburg, Russia, Vaschillo systematically studied relationships between breathing and heart rate, using a “transfer function analysis,” whereby the interplay between two oscillations is studied at different frequencies – i.e., different respiration rates. The maximum heart rate oscillations at respiratory frequency occurred at approximately 0.1 Hz (six breaths per minute), the one frequency at which heart rate oscillates with breathing at a 0° phase relationship, i.e., exactly in phase. Thus breathing at this frequency produces both the highest amplitude of RSA and the most efficient gas exchange.

It should also be noted that respiratory-induced changes in HRV may function as a positive feedback loop, spiraling further increases in HRV, by feedback from the heart to the central nervous system, through the vagal afferent system, as described below.

These results also suggest that, where HRV biofeedback produces a 0° phase relationship between heart rate and breathing, conditions requiring better gas exchange efficiency could show improvement. Consistent with this, there is evidence for better athletic performance after training in HRV biofeedback (Strack, 2003; Shaw, 2011; Paul and Garg, 2012) and that HRV biofeedback may help improve breathing symptoms and quality of life among patients with emphysema (Giardino et al., 2004).

## PHASE RELATIONSHIPS BETWEEN HEART RATE AND BLOOD PRESSURE

Vaschillo’s early studies also showed systematic changes in phase relationships between heart rate and blood pressure, when the system was stimulated at various frequencies. For each person, he found, there was a specific frequency where heart rate changes per unit change in blood pressure were greatest. This frequency varied from individual to individual, but was ∼0.1 Hz. (In later research we refined the average frequency for highest heart rate oscillations to be at about 0.09 Hz, or 5.5 breaths per minute, with breath duration of about 11 s; Vaschillo et al., 2002.) When he examined phase relationships between heart rate and blood pressure, he found that, at this frequency, heart rate and blood pressure oscillated in a 180° phase relationship: i.e., completely out of phase, such that blood pressure began falling as soon as heart rate began rising, and blood pressure began rising as soon as heart rate began falling. This phase relationship strongly suggested that the mechanism for the high-amplitude heart rate oscillations was the baroreflex.

## THE BAROREFLEX

The baroreflex is a reflex mediated by blood pressure sensors in the aorta and carotid artery that help modulate blood pressure fluctuations (Eckberg and Sleight, 1992). Baroreceptors in the walls of these arteries detect stretching of the arteries as blood pressure increases. When blood pressure increases, the baroreflex causes immediate decreases in heart rate. As blood pressure falls, the baroreflex causes immediate increases in heart rate. Thus, when the system is stimulated at the specific frequency causing maximum heart rate oscillations and a 180° phase relationship between heart rate and blood pressure, effects of the stimulator are compounded by effects of the baroreflex. As external stimulation causes heart rate to rise, it also causes blood pressure to fall, thus causing an additional stimulus for heart rate to rise further; and as external stimulation causes heart rate to fall, it also causes blood pressure to rise, thus causing an additional stimulus for heart rate to fall further.

Because of the 0° phase relationship between heart rate and breathing at approximately the same frequency that external stimulation causes maximal stimulation to the baroreflex, breathing becomes a natural way to provide external stimulation to increase HRV. Conversely, each breath then stimulates the baroreflex. We have found large increases in baroreflex gain (number of beats per minute change in heart rate per 1 mm Hg change in blood pressure) during HRV biofeedback: i.e., the baroreflex operates more strongly (Lehrer et al., 2003). When HRV biofeedback is practiced twice daily at home over about a 3 month period, we also find increases in *resting* baroreflex gain (i.e., before people start practicing biofeedback in a given session; Lehrer et al., 2003). This demonstrated neuroplasticity in the baroreflex, and suggested that regular exercise of the reflex rendered it stronger. It also suggested that various conditions affected by blood pressure lability and baroreflex control may be affected by HRV biofeedback. Thus, there is a growing body of evidence that a course of HRV biofeedback can help hypertensive patients lower their blood pressures (Nolan et al., 2010; Wang et al., 2010; Lin et al., 2012).

Pathways of baroreflex neural control suggest other possible HRV biofeedback applications. The baroreflex is mediated through the nucleus tractus solitarius, located in the brain stem (Raven et al., 1997; Rogers et al., 2000; Polson et al., 2007; Arnold et al., 2009). This center communicates directly with the amygdala, a center for emotional control, in a pathway extending through the insula (Volz et al., 1990; Henderson et al., 2004). Perhaps it is for this reason that various studies have shown positive HRV biofeedback effects for treating anxiety and depression (Karavidas et al., 2007; Reiner, 2008; Siepmann et al., 2008; McCraty et al., 2009; Nada, 2009; Zucker et al., 2009; Henriques et al., 2011; Tan et al., 2011; Patron et al., 2013).

## THE BAROREFLEX, HRV, AND RESILIENCE

There is a large amount of evidence that people are more resilient – physically and emotionally – when HRV oscillation amplitudes are higher and more complex. Greater complexity, as measured by various calculations of fractal entropy, suggest the operation of multiple regulatory feedback loops. One can think of this as the system having multiple backup systems to regulate the body, and finely tune it to environmental and internal need. Thus individuals with low HRV have generally impaired function: i.e., they are physically (Volz et al., 1990; Henderson et al., 2004) or emotionally (Friedman and Thayer, 1998; Gorman and Sloan, 2000; Carney and Freedland, 2009; Kemp et al., 2010) sick, are older (Fukusaki et al., 2000; Valentini and Parati, 2009; Nunan et al., 2010; McNarry and Lewis, 2012), are less aerobically fit (De Meersman, 1993; Hautala et al., 2003; McNarry and Lewis, 2012; Boutcher et al., 2013), and, when greatly physically compromised, at greater risk of dying (Kudaiberdieva et al., 2007; Laitio et al., 2007; Chan, 2008; Politano et al., 2008; Ranpuria et al., 2008; Stein, 2008; Ahmad et al., 2009; Thayer et al., 2010; Christensen, 2012; Handa et al., 2012; Huikuri and Stein, 2013). Total HRV in these studies generally is measured by the standard deviation of normal beat-to-beat intervals, i.e., intervals controlled by central nervous system input to the sinoatrial node of the heart, rather than by abnormal cardiac function). People with simpler patterns of HRV appear to be similarly compromised (Otsuka et al., 1997; Srinivasan et al., 2002; Yeragani et al., 2002; Skinner et al., 2008a,b, 2009; Huikuri et al., 2009). For this reason, HRV is often seen as a measure of physical and emotional resilience. We have found that HRV biofeedback restores autonomic function that is suppressed when people are exposed experimentally to inflammatory cytokines (Lehrer et al., 2010). *All* frequencies are suppressed by these cytokines, much as happens when we catch the flu or are subjected to another inflammatory condition.

## RESONANCE

It is a physical principle that all oscillating feedback systems with a constant delay have the characteristic of resonance. A resonant system is one that, when stimulated, produces high-amplitude oscillations at a single frequency, recruiting or overshadowing other frequencies, to produce a sine wave oscillation with very high-amplitude (Başar, 1998). An example of this from everyday life is the so-called Larsen effect, where a high pitched squeal at a single frequency results from placing a microphone in front of a speaker, and stimulating the system with sound (Weaver and Lobkis, 2006).

The same thing appears to happen in the cardiovascular system. The constant delay appears to be caused by amount of blood in the system, although, theoretically, flexibility, and diameter of the blood vessels also should play a role. We have found that taller people and men, who have a greater blood supply than, respectively, shorter people and women, have lower resonance frequencies (Vaschillo et al., 2006). That is, independently, stimulation at lower frequencies causes heart rate oscillations with the highest amplitude in taller people and men. Independently of height, age, and weight have no effect on resonance frequency, nor does experience with repeated system stimulation by HRV biofeedback.

If resonance occurs in the cardiovascular system at approximately 0.1 Hz, it should not occur exclusively in response to breathing. Rather, any source of rhythmic stimulation that affects the cardiovascular system should produce the same effect. This has, in fact, been found for rhythmic muscle tension (Lehrer et al., 2009; Vaschillo et al., 2011), and rhythmical presentation of emotion-inducing pictures (Vaschillo et al., 2008).

There also is evidence that resonance may occur at lower frequencies than 0.1 Hz. There is some evidence for resonance at about 0.02–0.03 Hz. While the source of this resonance is not completely known, it is known that the highest amplitudes of blood pressure oscillations are achieved when the system is stimulated at approximately this frequency (Vaschillo et al., 2002). Oscillations in this range are thought to be controlled primarily by the alpha sympathetic system, and related to oscillations in vascular tone, which also is affected by the baroreflex (Vaschillo et al., 2012). Thus, when blood pressure increases, the blood vessels dilate; when blood pressure falls, blood vessels constrict. This causes an oscillation in both blood pressure and vascular tone, but at a lower frequency than for the heart rate limb of the baroreflex, because dilation and constriction of blood vessels is a slower process than speeding or slowing the heart. Little is known about biofeedback training to stimulate the system in this frequency range. However, it is known that experienced Zen monks tend to breathe in this very slow range (Lehrer et al., 1999). Oscillations in this range are also thought to be involved in reflexes controlling thermoregulation (Fleisher et al., 1996; Thayer et al., 1997; Matsumoto et al., 1999).

## THE VAGAL AFFERENT PATHWAY

**FIGURE 2:**
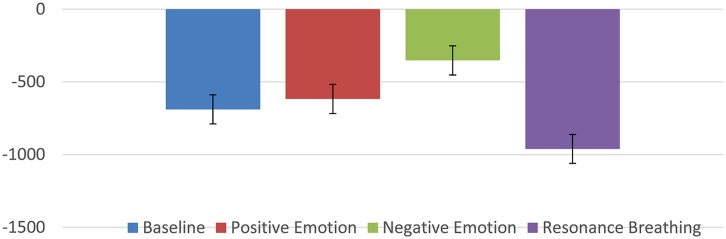
**N250 (in microvolts) evoked potential during various conditions**.

Several studies have reported that HRVB might be effective in reducing symptoms of depression and/or anxiety (Karavidas et al., 2007; Reiner, 2008; Siepmann et al., 2008; McCraty et al., 2009; Nada, 2009; Zucker et al., 2009; Henriques et al., 2011; Tan et al., 2011; Patron et al., 2013). These results led to speculation that some other mechanism might be at work beyond the baroreflex gains. One possible clue came from the recent research using vagal nerve stimulation for severe depression (and seizure disorders; Sackeim et al., 2001a,b; Nahas et al., 2005; Daban et al., 2008; George et al., 2008; Cristancho et al., 2011). An implanted electrical stimulation device is used to stimulate the vagal afferent pathways resulting in reduction of depressive symptoms. While this technique has not been subjected to larger random controlled trials, the preliminary pilot studies do raise interesting possibilities. It is known that the vagal afferent pathways affect brain areas known to be involved in affect regulation and mood (locus coeruleus, orbitofrontal cortex, insula, hippocampus, and amygdala; Grundy, 2002). In addition, there has been speculation that stimulation especially of the sub-diaphragmatic pathways through slow deeper breathing techniques might be stimulating these same pathways and thus having an effect on depressive/anxiety symptoms (Brown and Gerbarg, 2005a; Porges, 2011; Brown et al., 2013). We discovered some literature that offered the possibility that these pathways could be investigated using a technique called heart period evoked potentials (HEPs). Schandry and his colleagues had used this procedure to study interoception some years ago (Schandry, 1981, 2003; Schandry et al., 1986; Montoya et al., 1993; Critchley et al., 2004; Pollatos et al., 2005a,b; Gray et al., 2007). The HEP is a unique version of the usual evoked response (ERP) in that the R-wave of the ECG is used as a signal rather than being filtered out. Each heart beat then creates a large electrical signal to the brain which can be measured with surface electrodes. The above mentioned studies discovered that those subjects that had better interoception (ability to perceive their heart rates) produced a larger evoked potential presumably by way of the vagal afferent system. So if HRVB is, in fact, stimulating beneficial brain structures, we might see it reflected in the HEP. Thus far, two studies have supported this idea. In the first (MacKinnon et al., 2013), we examined the HEP waveform (it’s called an N250 because it produces a negative deflection at about 250 ms) at baseline in a resting state, during a negative emotion induction, during a positive emotion induction, and during a slow resonance breathing period. **Figure 2** shows the results.

As can be seen, the slow breathing condition was significantly more negative than the other conditions.

**FIGURE 3:**
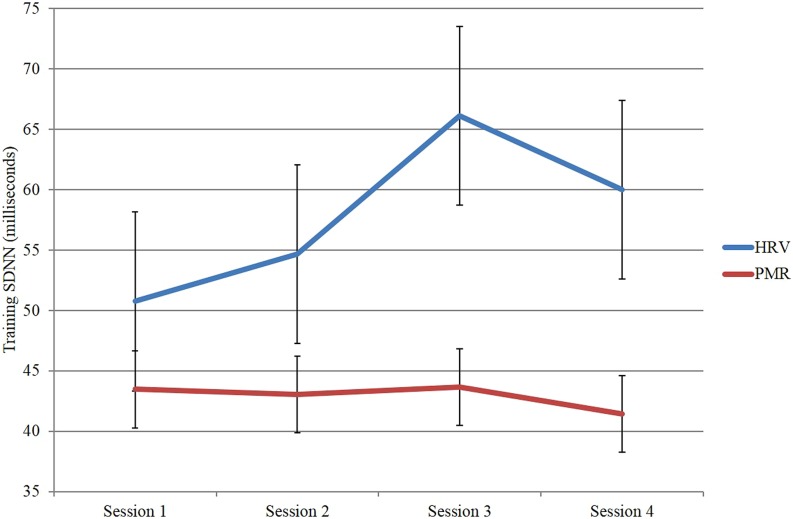
**Heart rate variability as measured by SDNN across sessions for the heart rate variability biofeedback group compared to the EMG/relaxation group**.

In a second study (Huang et al., 2014), we trained a group of 12 participants in HRVB over four sessions and compared them to a group of 13 participants who received EMG/relaxation training again over four sessions. As expected the HRVB group improved their HRV substantailly whereas the EMG comparison group did not (see **Figure 3**).

**FIGURE 4:**
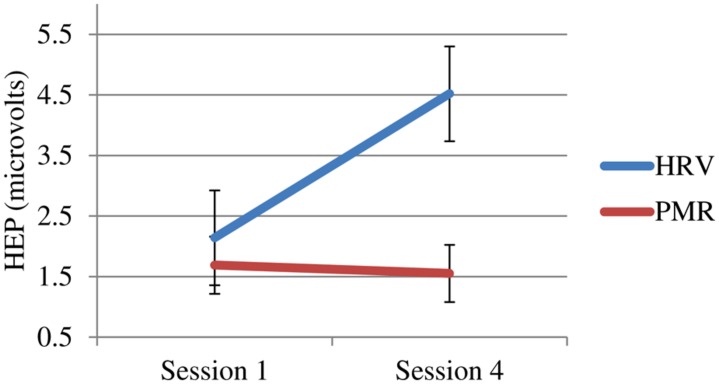
**Heartbeat event related potential (HEP) at 250 μs for both groups pre vs. post-training (sign reversed)**.

More importantly, only the HRVB group showed changes in their HEP (see **Figure 4**).

These results support our speculations and those of Brown and Gerbarg:

“… voluntary control of breath patterns can affect ANS functions via vagal afferents to brainstem nuclei (nucleus tractus solitarius, parabrachial nucleus, locus coeruleus)…Our neurophysiologic model postulates that vagal afferents activate hypothalamic vigilance areas and enhance and enhance attention and alertness, whereas pathways through the thalamus quiet frontal cortical activity and reduce anxious worrying” (Brown and Gerbarg, 2005a,b, p. 713).

Of course, there are many other possible explanations for why the complex process that occurs with HRVB might reduce depression and/or anxiety (distraction, self-efficacy, etc.). However, HEP method might give us a tool to disentangle possible mechanisms of the intervention.

## OTHER POTENTIAL MECHANISMS FOR THERAPEUTIC EFFECTS

Because HRV biofeedback is apparently helpful to conditions involving various physiological systems (pain, anxiety, depression, COPD, blood pressure control, athletic performance, etc.), it is probable that a number of mechanisms are involved in various effects, in addition to baroreflex stimulation and effects of vagal afferents. Most of them have received little empirical attention, but they all deserve investigation at this point. Possible mechanisms include:

### EFFECTS OF VAGAL EFFERENTS

Parasympathetic activity is usually a component in the “relaxation response.” Stimulation of parasympathetic reflexes by HRV biofeedback may produce body autonomic activity characteristic of relaxation, and thus directly counter stress effects. One way this may occur is a mechanism that has been labeled “Accentuated Antagonism.” “Vagal ‘tone’ predominates over sympathetic tone at rest. Under normal physiological conditions, abrupt parasympathetic stimulation will inhibit tonic sympathetic activation and its effects at rest and during exercise. This response is known as ‘accentuated antagonism’ (Olshansky et al., 2008, p. 863)”. Presumably this aspect of the parasympathetic efferent system is strengthened with HRVB training. This may be at play in inhibiting sympathetic output to myofascial trigger points (Hubbard and Berkoff, 1993; Gevirtz et al., 1996; Hubbard, 1998). The work of the Aziz group in London (Hobson et al., 2008) has also demonstrated that slow breathing almost immediately prevents esophageal pain thresholds from dropping dramatically when acid is introduced to the stomach.

### INCREASED GAS EXCHANGE EFFICIENCY

Because of the 0° phase relationship between breathing and heart rate during resonance frequency breathing, improved gas exchange may help people with respiratory diseases, and may even decrease respiratory drive in people with stress-induced hyperventilatory reactions.

### MEDITATION EFFECT

Heart rate variability biofeedback involves paying close attention to nuances in breathing. This is very similar to what is done in mindfulness meditation exercises. The pathway here would be primarily mental: i.e., one cannot simultaneously worry about various concerns of the day while concentrating on relaxed breathing.

### MECHANICAL STRETCHING OF AIRWAYS

Effects on asthma may occur indirectly through effects of stretching epithelial tissue in the lung by deep breathing. It is known that only a single deep inhalation during a methacholine challenge can decrease airway reactivity to methacholine in asthma patients (Pellegrino et al., 1996; Marchal et al., 2002).

### ANTI-INFLAMMATORY EFFECTS

It is known that the vagal system interacts closely with the inflammatory system, such that increases in vagus nerve traffic (usually produced by electrical vagal stimulation) are associated with decreases in serum levels of various inflammatory cytokines (Borovikova et al., 2000; Tracey, 2002). One study did find a decrease in C-reactive proteins among hypertensive patients treated with HRV biofeedback (Nolan et al., 2012). In another study, we experimentally exposed healthy subjects to an inflammatory cytokine, lipopolysaccharide (Lehrer et al., 2010). Usually both sympathetic and parasympathetic activity is blocked by lipopolysaccharide. Although no biofeedback-induced decreases in inflammatory cytokines were found, the autonomic effects of inflammation were greatly modulated, indicating that a greater resiliency was preserved among individuals given HRV biofeedback.

## CONCLUSION

In this paper we have tried to summarize possible mechanisms for the effectiveness of HRVB. We have been working under the assumption that increases baroreflex represented a viable marker of improved autonomic homeostasis or increased complexity. We have reviewed the evidence that led us to this conclusion, w*hich now are somewhat established* by our labs and others. We now speculate that in conjunction with this class of mechanisms, stimulation of the vagal afferent system may also play a role, especially in disorders of negative affectivity.

## Conflict of Interest Statement

The authors declare that the research was conducted in the absence of any commercial or financial relationships that could be construed as a potential conflict of interest.

## Footnotes

1 See Gevirtz (2013) for citations.
